# Treatment Success in *Trypanosoma cruzi* Infection Is Predicted by Early Changes in Serially Monitored Parasite-Specific T and B Cell Responses

**DOI:** 10.1371/journal.pntd.0004657

**Published:** 2016-04-29

**Authors:** María G. Alvarez, Graciela L. Bertocchi, Gretchen Cooley, María C. Albareda, Rodolfo Viotti, Damián E. Perez-Mazliah, Bruno Lococo, Melisa Castro Eiro, Susana A. Laucella, Rick L. Tarleton

**Affiliations:** 1 Hospital Interzonal General de Agudos Eva Perón, Buenos Aires, Argentina; 2 Center for Tropical and Emerging Global Diseases, Athens, Georgia, United States of America; 3 Instituto Nacional de Parasitología Dr. Mario Fatala Chaben, Buenos Aires, Argentina; FIOCRUZ - Minas, BRAZIL

## Abstract

**Background:**

Chagas disease is the highest impact parasitic disease in Latin America. We have proposed that changes in *Trypanosoma cruzi*-specific immune responses might serve as surrogate indicators of treatment success. Herein, we addressed in a long-term follow-up study whether cure achieved after treatment can be predicted by changes in non-conventional indexes of anti-parasite serological and T cell activities.

**Methodology/Principal Findings:**

*T*. *cruzi*-specific T cell responses, as measured by interferon-γ ELISPOT and *T*. *cruzi*-specific antibodies assessed by ELISA, hemagglutination and immunofluorescence tests as well as by a multiplex assay incorporating 14 recombinant *T*. *cruzi* proteins were measured in 33 patients at 48–150 months post-benznidazole treatment. Cure — as assessed by conventional serological tests — was associated with an early decline in *T*. *cruzi*-specific IFN-γ-producing T cells and in antibody titers measured by the multiplex serological assay. Changes in the functional status and potential of *T*. *cruzi*-specific T cells, indicative of reduced antigen stimulation, provided further evidence of parasitological cure following benznidazole treatment. Patients showing a significant reduction in *T*. *cruzi*-specific antibodies had higher pre-therapy levels of *T*. *cruzi*-specific IFN-γ- producing T cells compared to those with unaltered humoral responses post-treatment.

**Conclusions/Significance:**

Monitoring of appropriate immunological responses can provide earlier and robust measures of treatment success in *T*. *cruzi* infection.

## Author Summary

This study demonstrates that alterations in immunological parameters early after treatment with benznidazole in Chagas disease patients are predictors of treatment efficacy. Cure was associated with an early decline in *T*. *cruzi*-specific IFN-γ-producing T cells and in antibody titers and with high basal levels of *T*. *cruzi*-specific T cells.

## Introduction

Chagas disease is the highest impact parasitic disease in Latin America and the most common cause of infectious myocarditis in the world [1]. The goal of treatment of humans in the chronic phase of *Trypanosoma cruzi* infection is to prevent the development of heart disease and infection by via blood transfusion, congenital transmission and organ transplants [2]. However, treatment in adult chronic patients is not widely used mainly because of the lack of early metrics of treatment efficacy and the potential adverse effects of these therapeutics [3]. Several studies in adult patients with mild disease symptoms have demonstrated the clinical benefits of treatment with benznidazole [4,5]. However, the results of the recently published BENEFIT clinical trial [6] has raised questions about the benefits of benznidazole treatment in subjects with established cardiomyopathy, thus emphasizing that therapeutic interventions would have greatest benefit when delivered early in the infection.

The current criterion of a positive response to treatment is the complete loss of reactivity in serially performed conventional serological tests (ELISA, hemagglutination and immunofluorescence), as well as the lack of progression to more severe clinical conditions of Chagas disease. The decline in serologic titers using current standard tests is very slow, often requiring > 24 months for antibody titers in conventional tests to begin to fall; complete conversion to negative serology can take more than 10 years [4, 7–11]. Likewise, disease progression also occurs over decades and does not occur in all infected individuals [4, 5]. Consequently, the development of surrogate markers of treatment efficacy is needed for an early assessment of successful treatment and the evaluation of new therapeutic approaches in the chronic phase of *T*. *cruzi* infection.

CD4^+^ and CD8^+^ T cells derived from patients with chronic *T cruzi* infection have been shown to produce a variety of cytokines [12–18]. However recent studies using polychromatic flow cytometry revealed that CD4^+^ and CD8^+^ T cells with the capacity to produce only one cytokine (i.e. monofunctional T cells) in response to *T*. *cruzi* antigens is a common feature in adults with chronic Chagas disease [19–21]. Of note, monofunctional T cells are more prevalent in patients long-standing infections, generally accompanied by advanced cardiomyopathy [20,21], while polyfunctional T cells are often found in children who have shorter term infections [19]. This is consistent with the profile of pathogen-specific T cells in other infections where long-term antigen persistence maintains an active pathogen-specific T cell population but with increasing impairment of T cell function over time. This process known as immune exhaustion has been described for persistent viral, bacterial and protozoan infections [22–27] and is characterized by the loss of IL-2 production, cytokine polyfunctionality, as well as proliferative capacity followed ultimately, by defects in the production of IFN-γ, TNF-α, chemokines and degranulation potential [24]. Several other features of exhausted T cells, such as high expression of inhibitory receptors, a low expression of the IL-7 receptor and high dependence on the presence of antigen for T cell maintenance have been documented in patients with very long-term *T*. *cruzi* infections [20, 28–30].

We have proposed that changes in *T*. *cruzi*-specific IFN-γ-producing T cells [30] and declines in parasite-specific antibodies as measured by the non-conventional multiplex method might serve as surrogate indicators of treatment success, as determined in a 3-5-year post-treatment follow-up study in chronic Chagas disease patients [7, 30]. We hypothesize that treatment decreases parasite load, thus diminishing the antigen necessary to continually activate *T*. *cruzi*-specific T cells and B cells. In patients successfully cured of the infection, a stable change in T and B cell phenotype and activation, in line with antigen-independent immunological memory, would be expected.

In this study, the evolution of the functional profile of *T*. *cruzi-*specific T cells and of the humoral immune response to multiple *T*. *cruzi* antigens, in association with changes in conventional serological tests — an accepted marker of treatment efficacy — was assessed in 33 subjects chronically infected with *T*. *cruzi* over ~8 years following treatment with benznidazole.

We present evidence that cure — assessed by conventional serological tests — achieved many years after treatment with benznidazole was associated with an early decline in *T*. *cruzi*-specific IFN-γ-producing T cells, and in antibody titers measured by the multiplex assay. Changes in the activation status and potential of *T*. *cruzi*-specific T cells, indicative of reduced antigen stimulation, provided additional evidence of parasitological cure following benznidazole treatment. These results further support the case for using immunological markers as indicators of treatment efficacy in *T*. *cruzi* infection.

## Methods

### Selection of study population

*T*. *cruzi*–infected adult volunteers aged 23–54 years were recruited at the Chagas Disease Section of Hospital Interzonal General de Agudos Eva Perón, Buenos Aires, Argentina. *T*. *cruzi* infection was determined by indirect immunofluorescence assay, hemagglutination, and enzyme-linked immunoassay techniques [31] performed at the Instituto Nacional de Parasitologia Dr. Mario Fatala Chaben, Buenos Aires, Argentina. Chronically infected subjects were evaluated clinically and stratified according to a modified version of Kuschnir grading system [7, 32]. Individuals in group 0 had normal electrocardiograph, normal chest radiograph, and normal echocardiograph findings (n = 27, median age = 39 years, range = 23–54 years), and subjects in group 1 had normal chest radiograph and echocardiograph findings but abnormal electrocardiograph findings (n = 6, median age = 42 years, range, 30–50 years). Treatment consisted of benznidazole, 5 mg/kg per day for 30 days [5–9]. Clinical, serological and immunological analysis was performed prior and after treatment. Patients enrolled in this study did not change the clinical status during the follow-up period. This protocol was approved by the institutional review boards of the Hospital Interzonal General de Agudos Eva Perón, Buenos Aires, Argentina and the University of Georgia, GA, USA. Signed informed consent was obtained from all individuals before inclusion in the study.

### Collection of peripheral blood mononuclear cells (PBMCs) and serum specimens

PBMCs were isolated by density gradient centrifugation on Ficoll-Hypaque (Amersham) and were cryopreserved in a solution of 20% dimethylsulfoxide in heat-inactivated fetal calf serum for later analysis. Blood to be used for serum analysis was allowed to coagulate at 4°C and centrifuged at 1000 *g* for 15 min for sera separation.

### IFN-γ and interleukin (IL)–2 enzyme-linked immunosorbent spot (ELISPOT) assays

The number of *T*. *cruzi*–specific IFN-γ– and IL-2–secreting T cells was determined by ex vivo ELISPOT using a commercial kit (ELISPOT Human IFN-γ or IL-2 ELISPOT Set; BD), as described elsewhere [33]. To avoid inter-experiment variations, assays were conducted with paired samples from different time points assayed in the same experiment. Each time point was assessed 1–3 times.

### Monoclonal antibodies

mAb anti-CD3-fluorescein isothiocyanate (FITC), anti-CD134 (FITC), anti-IFN-γ (FITC), anti-CD25 (PE), anti-CD154 (PE), anti-CD3-peridinin chlorophyll protein (PerCP), anti-CD4 (PerCP), anti-CD27-allophycocyanin (APC), anti-TNF-α (APC) and anti-CCR7-phycoerythrin-Cy7 (PE-Cy7) and anti-CD4 (APC-Cy7) were purchased from BD Pharmingen, USA.

### Polyfunctionality of peripheral blood mononuclear cells and phenotyping of total T cells

PBMCs isolated from *T*. *cruzi*-infected subjects were stimulated with 15 μg/ml *T*. *cruzi* amastigote lysate or medium alone in 48-well plates at 37°C in a CO2 incubator for 16–20 h. Ten micrograms of brefeldin A per ml was added to the samples for the last 6 h of incubation. After stimulation, PBMCs were removed from the plates and stained for cell surface markers followed by fixation and permeabilization with cytofix/cytoperm and intracellular staining with a combination of monoclonal antibodies specific for IFN-γ, TNF-α and CD154 (CD40L). In order to confirm that cytokine/co-stimulation expression was derived from T cells, antihuman CD3 was added in polyfunctional staining assays in combination with CD4, IFN-γ and TNF-α or CD4, IFN-γ and CD154, respectively. Typically, 500,000 lymphocytes were acquired on a FACScalibur (Becton Dickinson Immunocytometry Systems, USA) and analyzed using FlowJo software (TreeStar, Inc., USA). Lymphocytes were identified based on their scatter patterns and CD4 expression for the combination of IFN-γ, TNF-α and CD154; and based on scatter patterns as well as CD3 and CD4 expression for the combination of IFN-γ and TNF-α or IFN-γ and CD154. Boolean combination gating was then performed to calculate the frequencies of expression profiles corresponding to the seven possible combinations of functions by using FlowJo. After subtracting the background values, the proportions of the different subsets were expressed as percentages of total cytokine or CD154-positive cells. Responses to the *T*. *cruzi* lysate were considered positive, for any particular subset, if the frequency of cytokine/CD154-positive T cells was threefold higher than the frequency in medium alone and above 0.07% of total CD4^+^ T cells, since the limit of detection was set at 0.01%.

### Multiplex serodiagnostic assay

Serum specimens were screened for antibodies reactive to a panel of 14 recombinant *T*. *cruzi* proteins in a Luminex-based format, as previously described [34]. Serological responses to each individual *T*. *cruzi* protein were considered to have decreased during the study period if the mean fluorescence intensity in at least one recombinant protein declined by 50% relative to that of the time 0 (pretreatment) sample assessed concurrently.

### Statistical analysis

Comparisons on the changes in *T*. *cruzi*-specific antibodies after treatment, measured by conventional serological tests, were performed using the Mann-Whitney U test. T cell responses at different time points were compared by Friedman range test. Comparisons of proportions were performed by use of the χ^2^ test and Fisher’s exact test. Differences were considered to be statistically significant at P<0.05.

## Results

### Long-term monitoring of T cell responses after treatment with benznidazole in chronic Chagas disease

**Fig 1:**
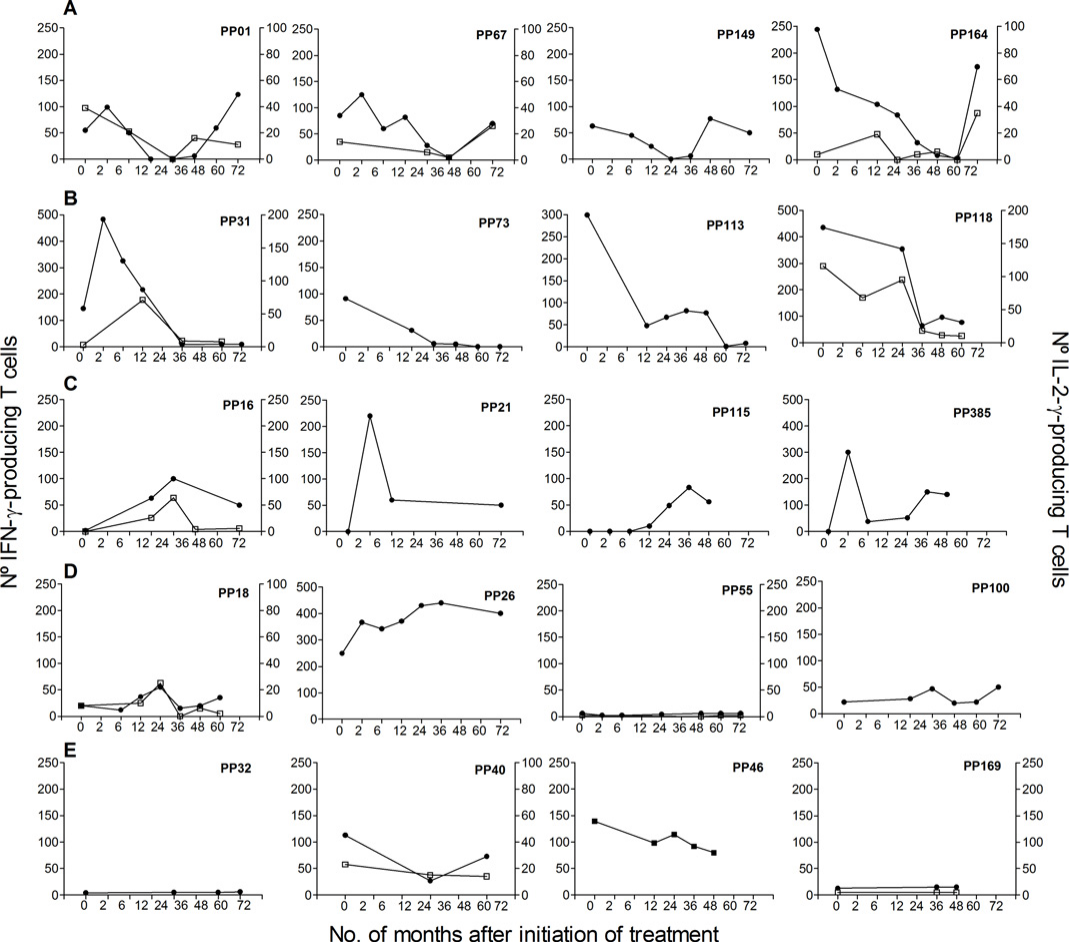
Monitoring of IFN-γ and IL-2 production in subjects with chronic Chagas disease and treated with benznidazole. IFN-γ– or IL-2-producing T cells were measured at different time points after benznidazole treatment or enrollment (for untreated subjects). Plots exhibit representative data for single subjects with different kinetics of T cell responses after benznidazole-treatment. Time 0 indicates the assay point just prior to benznidazole treatment. A) Parasite-specific T cell responses became undetectable after treatment and show a rebound thereafter. B) Parasite-specific T cell responses decreased after treatment. C) Previously undetectable cytokine-producing T cells prior to treatment became detectable after treatment. D) The frequencies of cytokine-producing T cells did not change relative to pretreatment. E) Monitoring of T cell responses in untreated subjects.

We have previously shown in a 3–5 year follow-up study that the frequency of peripheral IFN–γ-producing T cells responsive to *T*. *cruzi* antigens declined as early as 12 months after treatment with benznidazole and subsequently became undetectable in a proportion of treated subjects [30]. In some cases, these individuals with declining T cell responses experienced rebounds in parasite-specific T cell responses several years after treatment. Additionally, some subjects had undetectable IFN-γ-producing T cells (i.e. below background levels) prior to treatment that became detectable after treatment, whereas the frequencies of IFN-γ-producing T cells did not change relative to pretreatment in a fourth subset of subjects [30]. Herein, we report a 4-12-year follow-up (median 8 years) of humoral and cellular T cell responses in 33 of these subjects. All subjects for which IFN-γ ELISPOT responses fell below the level of detection between 12–36 months following treatment with benznidazole (n = 12) showed a later rebound in IFN-γ-producing T cells (i.e. range 24–72 months post-treatment) [Table 1, Group 1; Fig 1A]. In contrast, in the remaining subjects, T cell responses did not change significantly during long-term follow up (Table 1,Groups 2–4; Fig 1B–1D), Likewise, IFN-γ ELISPOT responses are relatively stable in 6 untreated subjects with a 48–60 month-follow-up (Fig 1E).

**Table 1:** Changes in IFN-γ ELISPOT T cell responses specific for *T*. *cruzi* antigens in long-term follow-up of chronic Chagas disease patients treated with benznidazole.

Patient group	N	Clinical stage		IFN-γ-ELISPOT responses	IFN-γ-ELISPOT responses
				at 36 months post-treatment	48–72 months post treatment
		G0	G1	relative to pretreatment ^A^ [30]	
1	12	9	3	Became undetectable	Rebound
2	6	5	1	Decreased	Unchanged
3	5	4	1	Became detectable ^B^	Unchanged
4	8	7	1	Unchanged	Unchanged

Note^. A^ Ages are not significant different among groups.

^B^ IFN-γ-producing T cells are undetectable prior to treatment and became detectable following treatment.

### Evolution of *T*. *cruzi*-specific antibodies in relation to changes in T cell responses in benznidazole-treated subjects

**Fig 2:**
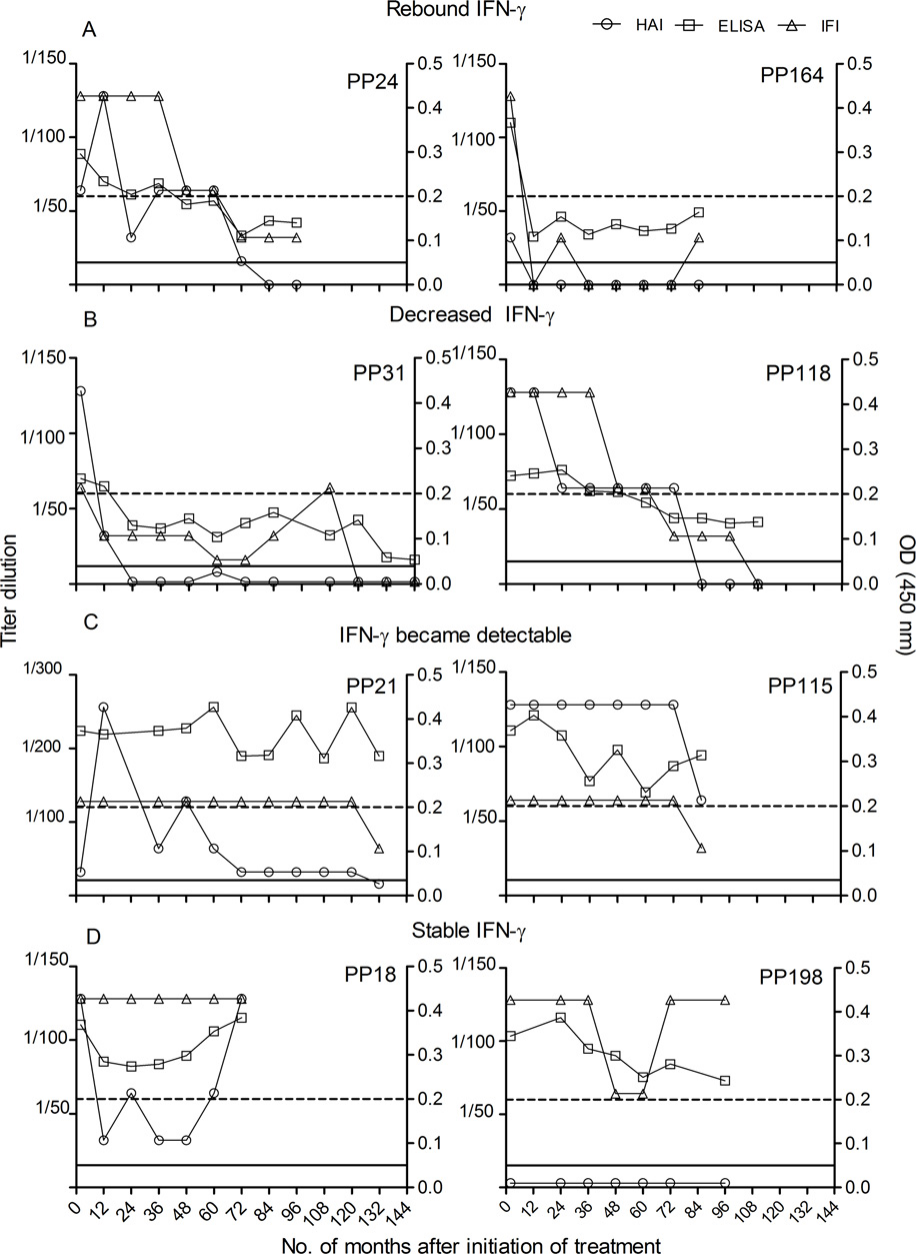
Evolution of *T*. *cruzi*-specific humoral responses in relation to changes in T cell responses after treatment with benznidazole. *T*. *cruzi*-specific humoral responses were measured at different time points after benznidazole treatment. by enzyme-linked immunosorbent assay (ELISA), indirect hemagglutination (IHA) and indirect immunofluorescence (IFI). Each panel exhibits representative humoral responses for single patients with different kinetics of IFN-γ producing T cells after benznidazole treatment. A) Parasite-specific T cell responses became undetectable after treatment and experienced a rebound thereafter. B) Parasite-specific T cell responses decreased after treatment. C) Undetectable cytokine-producing T cells prior to treatment became detectable after treatment. D) The frequencies of cytokine-producing T cells did not change relative to pretreatmentTime 0 indicates the assay point just prior to benznidazole treatment. Broken lines indicate the cut-off value for ELISA assays; full lines indicate the cut-off value for IHA and IFI assays.

**Fig 3:**
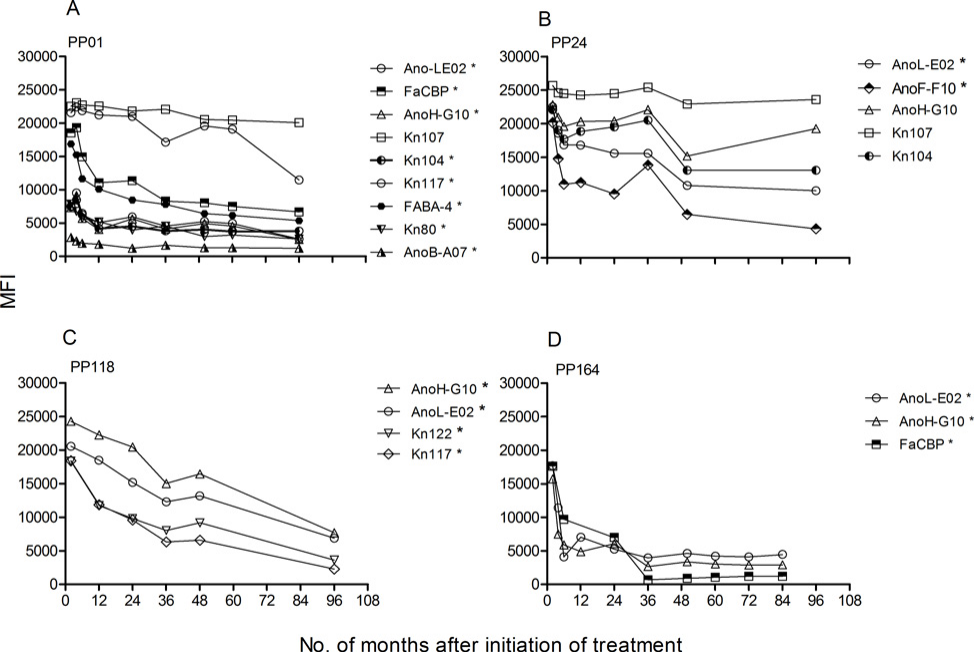
Multiplex serological analysis of *T*. *cruzi*-specific antibodies after long-term follow-up of chronic Chagas disease patients treated with benznidazole. Serum specimens obtained at the indicated time points were screened using a bead array-based multiplex serological assay with recombinant *T*. *cruzi* proteins, as described in Material and Methods. (A-D) Representative examples of the monitoring of *T*. *cruzi*-specific antibodies by the multiplex assay in subjects in which *T*. *cruzi*-specific IFN-γ-producing T cells became undetectable after treatment and show a rebound thereafter. Mean fluorescence intensity (MFI) for reactive proteins is shown. Time 0 indicates the assay point just prior to benznidazole treatment. (*) Indicates a decrease in MFI higher than fifty percent compared with pre-treatment values.

**Fig 4:**
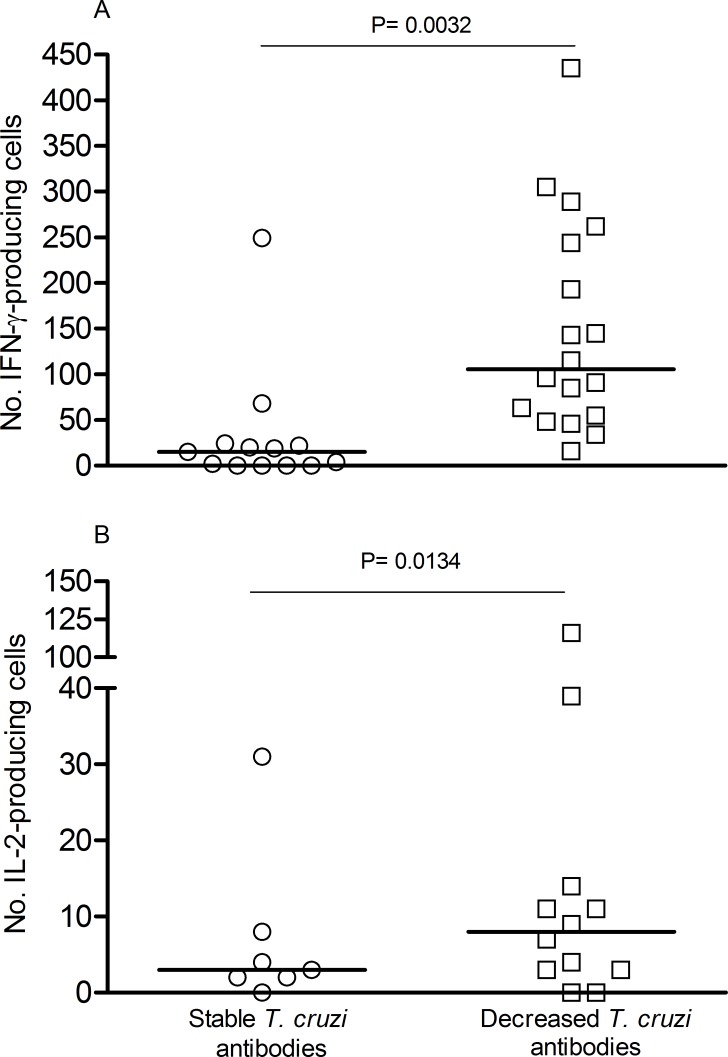
Levels of pre-therapy IFN-γ-or IL-2 secreting T cells in relation to the evolution to *T*. *cruzi*-specific humoral responses in Chagas disease patients treated with benznidazole. Treated subjects were grouped as those with stable or declining *T*. *cruzi*-specific antibodies post-treatment as measured by conventional serological tests and multiplex assays. Each dot represents the mean IFN-γ (A) and IL-2 (B) spot number of triplicate wells for each patient sample assessed. Spot counts with media alone were subtracted from *T*. *cruzi*–antigen stimulated spot numbers. Horizontal lines depict median values. Comparisons between groups were performed using the Mann-Whitney U test. P < 0.05 was considered as statistically significant.

Monitoring of *T*. *cruzi*-specific humoral immune responses assessed by the conventional serological tests, as well as by the multiplex assay that examines responses to 14 individual *T*. *cruzi* proteins [34], was conducted at least yearly following treatment with benznidazole. The levels of *T*. *cruzi*-specific antibodies measured by conventional serology significantly declined over time in subjects with decreased or rebounding IFN-γ-ELISPOT responses following treatment with benznidazole (Table 2 and Fig 2A and 2B) whereas antibody titers remained relatively stable in the other patient groups (Table 2, Fig 2C and 2D). Of note the seven patients who showed conversion from seropositive to seronegative–the standard metric of infection cure—on at least 2 of the 3 conventional serological tests were patient groups 1 and 2 (Table 2, Fig 2A and 2B). Conversion from seropositive to seronegative was observed on average >5 years post-treatment (24–96 months) and was sustained up to 12 years post treatment (Fig 2B, subject PP31). In concordance with conventional serology, a multiplex assay utilizing recombinant proteins from *T*. *cruzi* also revealed a higher rate of declining antibody titers among subjects with decreased or rebounding ELISPOT responses (Table 2, Fig 3A–3D). Seventeen out of nineteen patients with a rebound or a significant decrease in IFN-γ-producing T cells following treatment with benznidazole showed a fall in the levels of antibodies specific for one more recombinant proteins in comparison to 4 out of 13 in the group of patients in which T cell responses remained unchanged or became detectable after treatment (Table 2, Fig 3A–3D). Notably, the multiplex assay detected declines in antibody levels as early as 2–24 months post-treatment (Fig 3A–3D) while declines in conventional serologic tests were not evident until 24–48 months post-treatment (Fig 2A and 2B). Conversion from seropositive to seronegative by conventional serological tests can take up to 9 years to occur (Fig 2A and 2B). Thus, declines in *T*. *cruzi*-responsive IFN-γ-producing T cells and *T*. *cruzi*-specific multiplex-detected antibodies following benznidazole treatment preceded and were predictive of conversion to negative conventional serology, the accepted standard of treatment success. As previously reported [30], IL-2-producing T cells were low in chronically *T*: *cruzi*-infected subjects and changed in concert with IFN-γ T cell responses after treatment with benznidazole (Fig 2A–2E). Treatment success as measured by declining *T*. *cruzi*-specific antibody responses was not associated either with the age of subject at initiation of treatment or the baseline *T*. *cruzi*-specific antibody titers. However, subjects with declining antibody titers as a group had higher pre-treatment frequencies of IFN-γ- and IL-2 producing T cells as compared to patients who showed no change in humoral responses following treatment (Fig 4).

**Table 2:** Evolution of *Trypanosoma cruzi*-specific humoral immune responses according to changes in T cell responses during long-term follow-up of benznidazole-treated subjects.

Patient group	ELISPOT responses(from Table 1)				Changes in Serology		
		ELISA	IHA	IFI	No of subjects with seroconversion/total evaluated (%) ^A^	Multiplex serology (%) ^B^	Months of follow-up (range)
1	Rebound	0.0015 ^C^	0.0052 ^C^	NS	2/12 (17)	11/12 (92) ^E^	65–150
2	Decreased	0.0205 ^C^	0.0072 ^C^	NS	5/8 (63) ^D^	6/7 (86) ^F^ ^(*)^	48–132
3	Became detectable	NS	NS	NS	0/5	2/5 (40)	80–137
4	Unchanged	NS	NS	NS	0/8	2/8 (25)	48–96

^A^ No. of subjects with negative findings postreatment for 2 out of 3 or 3 out 3 conventional serological tests.

^B^ No. of subjects/total evaluated with a 50% decrease in mean florescence intensity for > 1 recombinant *Trypanosoma cruzi* protein in the14-protein multiplex panel.

^C^ P, antibody titers post-treatment compared with pretreatment values, by the Mann Whitney *U* test

^D^ P < 0.05 compared with group 4, by the Fisher exact test.

^E^ P < 0.01 compared with group 4, by the Fisher exact test.

^F^ P < 0.05 compared with group 4, by the Fisher exact test.

(*) No sample available for one patient.

ELISA, enzyme-linked immunosorbent assay; IHA, indirect hemagglutination; IFI, indirect immunofluorescence; NS, no significant change relative to pretreatment values.

### The cytokine and phenotype profile of rebound populations of *T*. *cruzi*-specific CD4^+^ T cells reflects absence of antigen stimulation

Since rebound in *T*. *cruzi*-specific T cells making IFN-γ was associated with declining serological titers, suggestive of a decreased presence of parasite antigen, we hypothesized that these *T*. *cruzi*-responsive T cells re-emerging long-term after treatment would result in enhanced functional capacity of *T*. *cruzi*-specific T cells.

**Fig 5:**
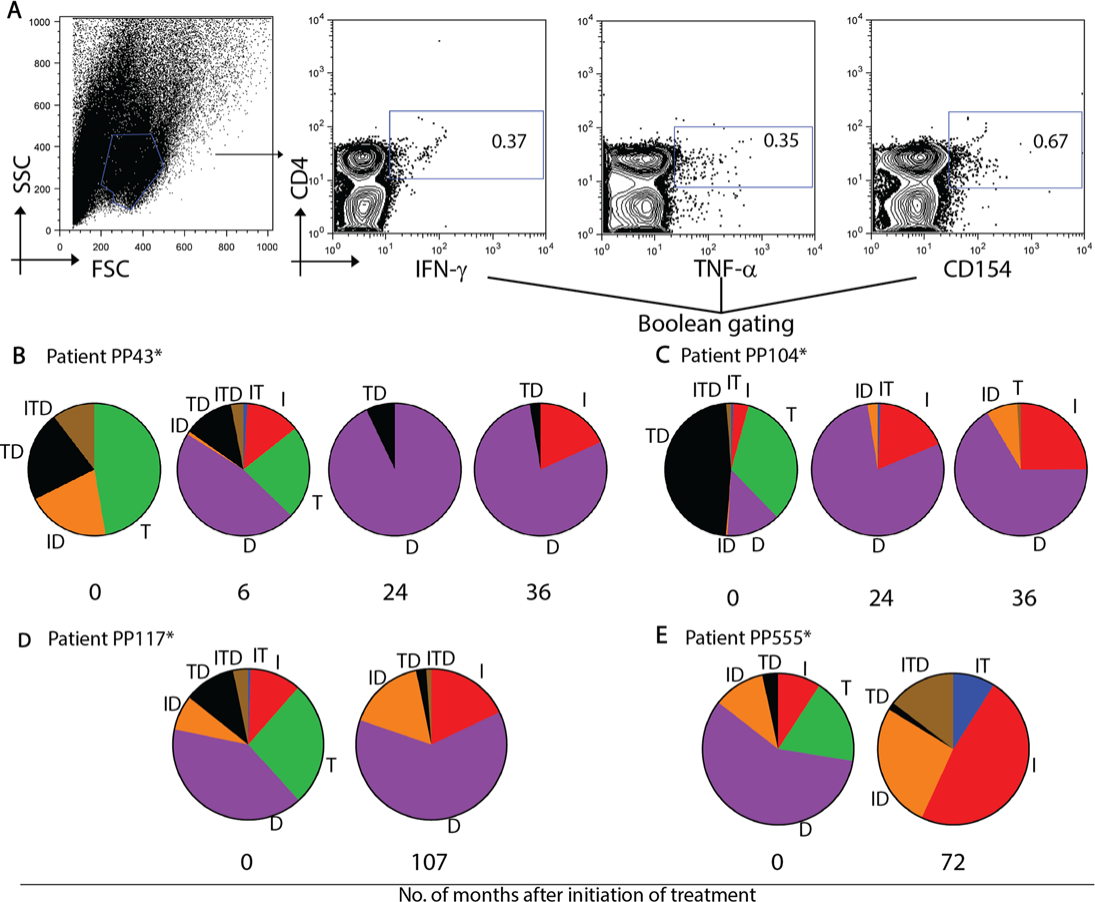
Longitudinal assessment of the functional capacity of CD4^+^ T cells responsive to *T*. *cruzi* antigens in chronic Chagas disease patients treated with benznidazole. PBMCs were stimulated with a *T*. *cruzi* lysate preparation or media alone and the expression of interferon (IFN)-γ, tumor necrosis factor (TNF)-α and CD154 was determined by polychromatic flow cytometry. (A) Lymphocytes were gated based on forward scattering (FSC) and side scatter (SSC), and CD4 T cells were then analyzed for IFN-, IL-2, and TNF-expression. Cytokine co-expression profiles were determined using the Boolean gating function of FlowJo software. Representative examples of four patients, PP43 (B); PP104 (C), PP117 (D) and PP555 (E) with rebound IFN-γ responses after treatment with benznidazole. Pies show the fraction of the total response that consist of CD4^+^ T cells positive for the different T cell subsets at different time points post treatment. (*) Indicates positive IFN-γ ELISPOT responses prior to treatment. (I) IFN-γ; (T), TNF-α; (D), CD154.

Group 1 subjects exhibited an increase in single CD4^+^CD54^+^ and CD4^+^IFN-γ^+^ T cells (Fig 5B–5D) coincident with a decrease in single CD4^+^TNF^+^ T cells (Fig 5B–5E) following treatment with benznidazole. Some subjects also showed an increase in dual IFN-γ^+^CD154^+^ T cells (Fig 5C–5E) or polyfunctional T cells with the ability to express IFN-γ; TNF-α; and CD154 (Fig 5E), These findings show that successful treatment resulted in a change of the functional profile of parasite-specific T cells with a restoration of the co-stimulatory function, generally impaired in chronic infections.

## Discussion

One of the primary drawbacks in treatment of chronic *T*. *cruzi* infections is the difficulty of assessing treatment efficacy [4, 35, 36], principally in the short term. In this study, we investigated if the early, post-treatment changes in *T*. *cruzi*-specific T cell and antibody responses, previously reported by our group [30], are predictors of treatment efficacy. To answer this question we compared these non-conventional immune assessments with the conversion from positive to negative conventional serology — the accepted standard of cure — in a longitudinal over ~8-year post-benznidazole treatment follow-up study. Our study revealed that cure—as determined by seronegative conversion by conventional serology—was strongly correlated with an early decline in both *T*. *cruzi*-specific T cells and in the levels of antibodies specific for a panel of *T*. *cruzi* antigens. Significant declines in IFN-γ-producing T cells and multiplex-monitored antibody responses post-treatment also preceded detection of reductions in anti-*T*. *cruzi* antibodies detectable by conventional serological tests. In contrast, subjects exhibiting stable T cell responses post-treatment were generally associated with unaltered conventional and multiplex-assessed humoral responses. Thus, this work identifies dependable and early markers of treatment efficacy in Chagas disease.

These results support and extend our previous studies [7] indicating the superiority of assaying responses to >10 recombinant proteins using a multiplex format over conventional serologic tests. Other studies have also demonstrated that the use of recombinant proteins as antigens can often detect changes in parasite-specific antibodies earlier than the complex *T*. *cruzi* antigen preparations normally used in many conventional tests [37, 38]. However, in 15 out of the 33 patients evaluated in this study slight or no changes in *T*. *cruzi*-specific humoral and cellular T cell responses were observed, suggesting a failure of treatment and confirming previous studies showing that benznidazole treatment is not uniformly successful curing *T*. *cruzi* infection [4, 6].

Some subjects with declining or negative anti-*T*. *cruzi* antibody levels and T cell responses experienced rebounds in T cell responses, prompting the question of whether these T cell reflected renewed antigen stimulation, and thus persistence of *T*. *cruzi* infection. However rebounding IFN-γ-producing T cells were associated with decreasing serological titers by both conventional and multiplex assays and two of the seven subjects who converted to negative conventional serology–the accepted standard of cure—exhibited this rebound in T cell responses. Therefore, it seems likely that these parasite-specific T cells in rebound responses are maintained in the absence of or very low levels of antigen, a characteristic of TCM. Such responses are evident in mice cured of *T*. *cruzi* infection by benznidazole treatment [8, 39, 40]. Herein, benznidazole treatment resulted in a different functional quality of CD4^+^ T cells with a prominent decline in single producers of TNF-α and an increase in either monofunctional or polyfunctional CD4^+^ T cells expressing CD154 after treatment. Several studies have shown that constant antigen stimulation during chronic infections might skew T cell responses to single TNF-α-producing T cells [41] and low CD154 expression [42, 43] which are restored after suppression of antigen load [41, 44].

Other studies have also shown that therapy with benznidazole in the chronic phase of the infection resulted in a shift toward a type- T cell profile profile [45–47]. Collectively, these findings further support that parasite persistence in chronic *T*. *cruzi* infection induces significant alterations in T cell function.

An interesting observation that deserves further investigation is that subjects who showed the greatest decrease in *T*.*cruzi*-specific antibodies following treatment also had on average higher baseline levels of IFN-γ-producing T cells compared with subjects with modest or no changes in humoral responses. Studies in the experimental models have suggested that the quality of the anti-*T*. *cruzi* immune response plays a role in the efficacy of benznidazole treatment [48–51]. Studies in larger patient groups and in experimental models are needed to confirm these findings.

This study validates the ability of appropriate and sensitive immunological tests to provide early evidence of treatment efficacy in chronic Chagas disease. Providing tools to not only monitor but to more rapidly predict treatment success or failure will facilitate the development of new and better therapeutic options in Chagas disease.
